# Machine Learning Models to Predict Protein–Protein Interaction Inhibitors

**DOI:** 10.3390/molecules27227986

**Published:** 2022-11-17

**Authors:** Bárbara I. Díaz-Eufracio, José L. Medina-Franco

**Affiliations:** DIFACQUIM Research Group, Department of Pharmacy, School of Chemistry, Universidad Nacional Autónoma de México, Avenida Universidad 3000, Mexico City 04510, Mexico

**Keywords:** chemoinformatics, computer-aided drug design, drug discovery, machine learning, protein–protein interaction

## Abstract

Protein–protein interaction (PPI) inhibitors have an increasing role in drug discovery. It is hypothesized that machine learning (ML) algorithms can classify or identify PPI inhibitors. This work describes the performance of different algorithms and molecular fingerprints used in chemoinformatics to develop a classification model to identify PPI inhibitors making the codes freely available to the community, particularly the medicinal chemistry research groups working with PPI inhibitors. We found that classification algorithms have different performances according to various features employed in the training process. Random forest (RF) models with the extended connectivity fingerprint radius 2 (ECFP4) had the best classification abilities compared to those models trained with ECFP6 o MACCS keys (166-bits). In general, logistic regression (LR) models had lower performance metrics than RF models, but ECFP4 was the representation most appropriate for LR. ECFP4 also generated models with high-performance metrics with support vector machines (SVM). We also constructed ensemble models based on the top-performing models. As part of this work and to help non-computational experts, we developed a pipeline code freely available.

## 1. Introduction

**Figure 1:**
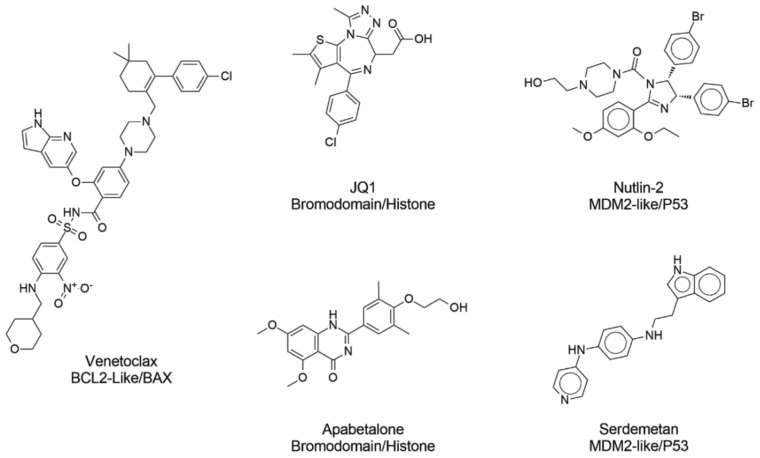
Examples of protein–protein interaction inhibitors currently approved for clinical use or under clinical trials.

In recent years, protein–protein interactions (PPI) have received increased attention as therapeutic macromolecular targets [1,2]. Designing PPI inhibitors is challenging because they have distinct molecular properties and occupy regions of chemical space differently from conventional small-molecule drugs [3]. For instance, PPI inhibitors are larger, are more hydrophobic, have more aromatic rings, and have distinct three-dimensional conformations compared to traditional small-molecule drugs [4]. In general, the properties of PPI inhibitors are significantly different from traditional drug candidates [5]. In recent years, several efforts have been made to develop PPI inhibitors [6], resulting in many PPI inhibitors in clinical trials [7] (Figure 1). An example is apabetalone, which has progressed to phase III clinical trials to prevent major adverse cardiovascular events in high-risk type 2 diabetes mellitus patients. Idasanutlin is being tested in clinical trials to treat neoplasia and leukemias [8]. Another representative example is Venetoclax, a BCL-2 inhibitor approved for clinical use to treat chronic lymphocytic leukemia and certain types of small lymphocytic lymphoma [9] (Figure 1). PPI inhibitors are a specific case of PPI modulators (modulation includes inhibition and stabilization).

Different compound databases of PPI inhibitors have been created. For example, TIMBAL [10], the Inhibitors of Protein–Protein Interaction Database (iPPI-DB) [11], Fr-PPIChem, and the databases reviewed therein [12]. This research was made possible thanks to the advances in cheminformatics techniques and the growing availability of PPI inhibitors data in the public domain [13]. Thanks to the availability of this data, it is possible to develop machine learning (ML) models [12]. The iPP-DB was one of the first efforts to develop available compounds with activity against 13 PPI targets: it was manually curated and includes 8,900 compounds. Examples of PPI targets included in this database are bromodomain proteins. Fr-PPIChem is a diverse library of 10,314 PPI-like inhibitors identified because of the implementation of artificial intelligence (AI) techniques. Of note, to the best of our knowledge, the rich data currently available for PPIs have not been fully used to develop predictive models.

AI and ML have enormous potential to revolutionize drug design and development [14,15]. ML is valuable because it uses pattern recognition algorithms to discern key features between molecules and properties and differentiate them [16]. The subfield of ML, deep learning (DL), uses artificial neural networks that adapt and learn from the vast amount of experimental data [14,17,18]. ML and DL have been successfully implemented in drug discovery programs [19,20]. Recently, Choi et al. employed principal component analysis and k-means clustering to classify and explore PPI inhibitors in chemical space based on drug-like physicochemical properties [4]. However, ML has not been reported to classify PPI inhibitors successfully based on molecular fingerprints.

This study aimed to generate ML predictive models to classify compounds as PPI inhibitors and make the code freely available to the scientific community, particularly medicinal chemists working with PPI inhibitors. The underlying hypothesis is that a large amount of structure–activity–relationships data of PPI inhibitors in public databases should facilitate the development of models with high predictive ability. As part of this work, we developed and implemented a pipeline script to automate the models’ training, save the output, and store the results in a report. The pipeline developed simplifies the process of different parameter settings in combination with a variety of molecular fingerprints of different designs, including extended connectivity radius four and six (ECFP4, ECFP6), Molecular ACCess system (MACCS) keys, and Atom Pairs. The details of the script are described in the Methods section.

## 2. Methods

### 2.1. Data Sets

*PPI inhibitors.* We assembled a compound database of PPI inhibitors from the IPP-Fr database [11] and ChEMBL_27 [21]. The database contains 2403 unique (non-duplicate) PPI inhibitors from 28 subfamilies (the subfamilies are summarized in Table S1 of the Supplementary Materials) that were set as the success case (positive set). All selected targets are reported as PPI on different databases such as HIPPIE [22,23]. The criterion to include a molecule in the set is the activity independent of its mechanism of inhibition. In this work, we consider a compound as “active” if the reported IC_50_, EC_50_, K_d,_, or K_i_ value is equal to or lower than 30 μM.

*Approved drugs.* A set with 2,403 small molecules approved for clinical use (except PPI inhibitors) obtained from DrugBank [24] was used to assemble a negative set to train the classification models.

### 2.2. Molecular Representations

Molecular representation is the core of chemoinformatics [25]. For this work, we used four fingerprints: ECFP4, ECFP6, MACCS keys, and Atom Pairs. The size of the feature vectors was 2048, 2048, 167, and 8718, respectively.

### 2.3. Machine Learning Models

To develop an ML model with the ability to identify a PPI inhibitor from the positive and negative set described in Section 2.1, the algorithms selected for implementation and training were those that include labeled data, known as supervised algorithms [26]. Three classification algorithms were implemented to develop a predictive model that classifies a molecule into a specific category (e.g., PPI inhibitor from non-PPI inhibitor): RF, LR, and SVM. For this work focused on the classification of PPI inhibitors, these three models were selected based on their well-known performance. However, several other models can be explored in future studies. The models were trained with different hyperparameters and initial setups described in Section 2.4. All algorithms employed in this work demonstrated their applicability in several chemical-related tasks [27,28,29], including the prediction of biological endpoints [30] and absorption, distribution, metabolism, excretion, and toxicity (ADMETox) properties [31]. Of note, as indicated in the Perspectives section (Section 4), the robustness of the ML initially proposed in this work was assessed with experimental data generated by our or other research laboratories working on the development of PPI inhibitors.

### 2.4. Training Models

#### 2.4.1. Data Proportions

Two proportions were used to assess which provided the best results: 80:20 and 70:30. However, other ratios can be explored in follow-up studies.

#### 2.4.2. Parameter Settings

(A) RF is an algorithm that generates many decision trees and then assembles their outputs [16]. The parameters explored for this algorithm are: the number of trees in the forest (100, 500, 1000) and gini and entropy as functions to measure the quality of a split. Details of the RF setup are summarized in Table S2 in the Supplementary Materials.
(B) LRG [32] is a linear classification model. In this model, the probabilities describing the possible outcomes of a single trial are modeled using a logistic function. Solver parameters have a major impact on results. Five different solvers included in scikit-learn were used: newton-cg, lbfgs, liblinear, sag, and saga (Table S3 in the Supplementary Materials).
(C) SVM [33] solves classification problems because of its ability to handle high-dimensional data using a kernel function [34]. In SVM, the kernel function is used to map data into high-dimensional space by finding an optimally separating hyperplane. For this study, four different kernels were used: linear, poly, rbf, and sigmoid (Table S4 in the Supplementary Materials).

### 2.5. The Automated Pipeline

The methodology established in solving problems similar to our study case involves a process containing several sequential and repetitive phrases. For this purpose, it was very useful to automate the workflow. Although there are tools such as KNIME [35] in this work, we developed our own pipeline to simplify common tasks such as training, model evaluation, and writing individual reports. Through the orderly execution of codes written using the python3 programming language and the python libraries: pandas, scikit-learn, numpy, and matplotlib. Of note, the pipeline elements can be used on any machine, computational cluster, or operating system and enable the code to be generated broadly and used to solve new problems in the future. These files were developed to make our code more readable and reproducible.

**Figure 2:**
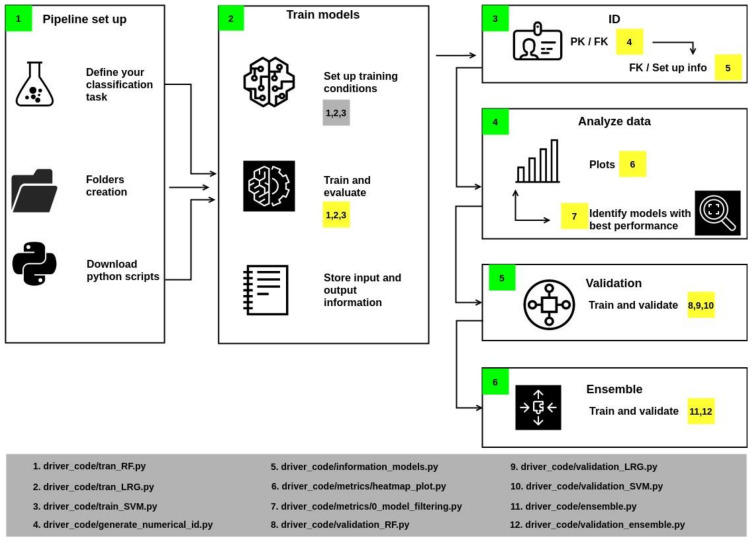
Workflow developed in this work to automatize the steps of training the classification methods, their evaluation, and output of the results.

The methodology implemented in the pipeline is divided into six sections (Figure 2): set-up, model training, identifier generation, data analysis, validation, and ensemble. Each section is described hereunder.

- In Section 1 of the workflow (Figure 2), it is necessary to create the folders and download the python files.
- In Section 2, some variables should be associated with specific values, such as allocating the files’ location. Training parameters must be assigned: once configured, run scripts 1, 2, and 3 to train the models. These scripts also calculate accuracy, precision, F1, and recall, which are metrics to describe the quality of the model’s predictions (metrics are computed from the test population). The script also stores the results in an individual report.
- Section 3 generates identifiers if the report’s name corresponds to the Primary Key. Then, a Foreign Keys is generated by the union of the initials of the algorithm and a numerical index. The results are stored in a JSON file.
- Section 4 includes a series of scripts to collect metrics values from reports and generate heatmaps. These plots contain information from those models whose values were greater than or equal to the statistical metric known as Q2 (i.e., the middle of the data set, also termed the 50th percentile).
- Section 5 implements cross-validation of models with values above Q2 with a k equal to 20. As a result, an output file is generated that reports the value of the mean and the deviation of accuracy.
- Section 6 performs the training and validation of a consensus model.

## 3. Results and Discussion

### 3.1. RF

All the trained models with RF had precision values higher than 0.91; the mean was 0.94, and the maximum value was 0.98 (Tables S5 and S8 and Figure S1 in the Supplementary Materials). The minimum and maximum accuracy values were 0.93 and 0.96, respectively. The maximum recall value (0.96) was lower than the maximum precision value but equal to the maximum accuracy value.

Models that shared maximum accuracy and precision values were RF4, RF6, and RF27, although these models did not share the maximum recall value. RF4 and RF6 were trained with ECFP4 but differed regarding the number of estimators and the number of decision trees employed. Both models had very similar performances. RF27 was trained with ECFP6. However, compared to RF4 and RF6, RF27 was slightly worse at predicting positives as measured by its recall value (0.93).

The RF models with good performance were generally trained with ECFP4, followed by ECFP6. In contrast, models with lower performance, e.g., lowest precision and accuracy values, were trained with MACCS Keys and Atom Pairs fingerprints.

Validation results obtained by cross-validation of model RF27 (Table S11 in the Supplementary Materials) suggest that the predictions are consistent across different test subsets (see results in Table 1). The training conditions of RF27 were selected to construct the ensemble model discussed in Section 3.4.

### 3.2. LRG

Eighty models with a different initial setup, such as fingerprint representation, dataset proportion, and solver configuration, were trained (evaluation metrics are summarized in Table S6 and the statistical values are in Table S9 and Figure S2 in the Supplementary Materials). Six of the twenty models trained from ECFP4 had a precision value equal to or higher than 0.95. The models with better precision were those trained with ECFP6. The minimum precision value was 0.76. These models have in common that they were trained with Atom Pairs employing a saga solver.

The maximum accuracy value (0.95) was obtained for five models: LRG21, LRG22, LRG23, LRG24, and LRG25, all trained with ECFP6. LRG21 and LRG22 were trained with newton-cg, RG25 was trained with a liblinear solver, and LRG23 and LRG24 were trained with lbfgs.

Models trained with Atom Pairs had a minimum accuracy value of 0.83 and had the lowest recall values.

There were no models that shared both maximum precision and recall values. However, models that share maximum accuracy and precision values, such as LRG22, LRG24, and LRG27, were trained with ECFP6.

LRG35, LRG36, LRG39, and LRG40 models had maximum recall values of 0.95. These models were trained with ECFP6.

LRG22, LRG24, and LRG27 were trained with the same proportion set: 0.20 for evaluations and 0.80 for training. LRG22 and LRG24 had the same number of true negatives in the confusion matrix: the value of true positives obtained with LRG22, LRG24, and LRG27 were 470 and reported maximum values for F1. Therefore, these models help identify negatives and positives. Based on good performance metrics values, the setup conditions of these models were employed as a reference to train a consensus model (see Section 3.4). The average accuracy, precision, and recall values were 0.91, 0.90, and 0.92, respectively. ECFP6 seems to be a good descriptor. In contrast, LRG models trained from Atom Pairs fingerprints did not yield good separations between positive and negative PPI inhibitors.

Table 1 summarizes the validation results of models selected for consensus analysis. The models chosen for the ensemble were LRG22, LRG24, and LRG27 which had equal accuracy, precision, and F1 values. Further details are summarized in Table S12 in the Supplementary Materials.

### 3.3. SVM

Sixty-four models with different initial setups were trained. The evaluation metrics are summarized in Table S7 and Figure S3 in the Supplementary Materials. The accuracy values obtained for SVM ranged between 0.95 and 0.62. The range of precision values was 0.99-0.59. The recall values ranged between 0.94 and 0.57.

The highest accuracy values were obtained by eight models with the maximum F1 value: SVM5, SVM6, SVM13, SVM14, SVM21, SVM22, SVM29, and SCM30. The eight models share the same kernel type, rbf, but contain different “class weights” and descriptors. Four models were trained with ECFP4 and four with ECFP6. SVM21 and SVM22 (trained withECFP6) had the highest precision and maximum accuracy values. In contrast, SVM13 trained with ECFP4 had the maximum recall value. These results suggest that for the PPI inhibitors used in this work, ECFP4 is better for training models with high precision and ECFP6 is more appropriate for training models with high recall values.

The mean accuracy values were 0.89, while the lowest value was 0.62, obtained by the SVM39, SVM40, and SVM47, models trained with MACCS Keys and a sigmoidal kernel.

The highest precision values were obtained with models trained with ECFP6 and the polynomial kernel. The lowest values were obtained for models trained with MACCS Keys and the sigmoidal kernel.

The model selected for consensus prediction (discussed in Section 3.4) was SVM22. This model had an accuracy and F1 values equal to the maximum statistical value for two metrics (Table S10 in the Supplementary Materials). The results of model validation are listed in Table 1. Uniquely, the models whose metrics were more significant than the value of Q2 were validated (Table S13). The result of this process is freely available at https://github.com/BarbaraDiazE/PPI_ML (accessed on 15 November 2022).

### 3.4. Consensus Prediction

In this part of the study, the primary purpose was to develop a consensus model to obtain predictions better than the individual models. The rationale is that a model trained from many single models should increase the model’s capability to discern between active and inactive PPI inhibitors.

After identifying the different models’ performance and their respective evaluation metrics (discussed in previous sections), we combined multiple models to improve the overall performance and yield a consensus prediction by generating an ensemble learning. We selected the five models summarized in Table 2 based on the following criteria: good values of evaluation metrics, the same proportion of training/test set, and the molecular representations employed during the training process. All models used in the ensemble were trained with ECFP6. Regarding the metrics, given the study’s primary goal, we decided to prioritize models with high precision and those that were more susceptible to making a correct prediction of success. Therefore, we selected models with good recall, prioritizing these models over others with high-balanced accuracy and high F1 values.

Ensemble 1 and Ensemble 2 (Table 2) had an accuracy value lower than RF27. However, Ensemble 1 predicted fewer FP and more TP, while Ensemble 2 was as good as RF27 predicting TP and a better detecting FP. All other models in Table 2 had accuracy records equal to both ensembles.

Regarding precision, SVM22, RF27, and Ensemble 2 had the maximum precision value, 0.98, in contrast to 0.95 of Ensemble 1. The confusion matrix indicated that SVM22 was not as good as RF27 and Ensemble 2 at identifying TP. Four of the five models employed in Ensemble 1 training shared the same recall value as Ensemble 2, while Ensemble 2 had a lower recall value.

Ensemble1 had the same metrics values as the LRG models employed in training exclusively registered different values on the confusion matrix (see Table 3). This result suggested that even when metrics were equal, Ensemble 1 was better at identifying TP than individual LRG models. Furthermore, Ensemble 2 was better than LRG models predicting TP.

Ensemble 2 was chosen to perform activity prediction because joined knowledge learned by individual models at the time obtained better predictive performance by deducing variation and generating a robust model [36]. This model also had lower accuracy standard deviation obtained by cross-validation (*k* = 20) with respect to the models reported in Table 1 (employed on training ensemble). This result suggested that although the performance metrics of the ensemble models are not better than those of the individual models, the results are more reproducible.

Table 3 summarizes predictions made with the models reported in Table 2 and both ensemble models exemplify the prediction results. Ensemble 1 made incorrect predictions in two instances in this focused case study, while the Ensemble 2 model only made one incorrect prediction. In Table 3, is noticeable that Venetoclax was a challenging case for several models, expect for RF27, SVM22 and Ensemble 2, that correctly predicted the compound as PPI inhibitors. This is likely due to the chemical descriptors used to train the models that could not accurately capture the unique structure of Venetoclax. This example further emphasizes the notable performance of Ensemble 2.

## 4. Conclusions and Perspectives

In this study, several ML algorithms were trained to develop predictive models to identify PPI inhibitors. Out of the different fingerprints used, the ones trained with ECFPs yielded, in general, the best results. Three trained models were selected to develop ensemble learning and perform a consensus prediction. Ensemble learning provides a prediction (PPI or not PPI inhibitor) by a voting decision instead of a single decision from one model. The outcome of this work is helpful because it presents predictive models that will aid data-driven decisions in future PPI inhibitor design projects.

As part of this study, we developed a code pipeline that facilitated the training of ML models to classify PPI inhibitors. The freely available code can be used with other data sets and molecular representations.

One of the main perspectives of this work is to conduct a prospective validation of the ML models by testing their ability to classify newly designed inhibitors made by medicinal chemists or published in the peer-reviewed literature. Another perspective is to update the database of PPI inhibitors periodically, e.g., on a bi-annual basis, to improve the performance of the individual and ensemble models. We also anticipate implementing an accessible webserver to facilitate the scientific community’s prediction of PPI inhibitors.

## Figures and Tables

**Table 1:** Validation results of selected individual and ensemble models.

ID	Accuracy Mean	Accuracy Std
RF27	0.957	0.014
LRG22	0.941	0.017
LRG24	0.941	0.017
LRG27	0.941	0.017
SVM22	0.958	0.015
Ensemble1	0.940	0.013
Ensemble2	0.956	0.010

**Table 2:** Metrics comparison. Single models vs. ensemble using ECFP6.

ID	Accuracy	Balanced Accuracy	Precision	Recall	F1	Confusion Matrix
RF27	0.96	0.96	0.98	0.94	0.96	[486 10] [32 401]
LRG 22	0.95	0.95	0.95	0.94	0.94	[470 23] [27 406]
LRG24	0.95	0.95	0.95	0.94	0.94	[470 23] [27 406]
LRG27	0.95	0.95	0.95	0.94	0.94	[470 23] [26 407]
SVM22	0.95	0.95	0.98	0.92	0.95	[484 9] [33 400]
Ensemble1	0.95	0.95	0.95	0.94	0.94	[471 22] [27 406]
Ensemble2	0.95	0.95	0.98	0.91	0.94	[486 7] [41 392]

**Table 3:** Models’ prediction for individual compounds.

	Real	RF27	RF22	LRG24	LRG27	SVM22	Ensemble1	Ensemble2
Venetoclax	Active	Active	Inactive	Inactive	Inactive	Active	Inactive	Active
Apabetalone	Active	Inactive	Active	Active	Active	Active	Active	Inactive
Idasanutline	Active	Active	Active	Active	Active	Active	Active	Active
JQ1	Active	Active	Active	Active	Active	Active	Active	Active
I-BET	Active	Active	Active	Active	Active	Active	Active	Active
Nutlin-2	Active	Active	Active	Active	Active	Active	Active	Active
Atorvastatin	Inactive	Inactive	Inactive	Active	Active	Inactive	Active	Inactive
Amoxicilin	Inactive	Inactive	Inactive	Inactive	Inactive	Inactive	Inactive	Inactive
Albuterol	Inactive	Inactive	Inactive	Inactive	Inactive	Inactive	Inactive	Inactive
Metformin	Inactive	Inactive	Inactive	Inactive	Inactive	Inactive	Inactive	Inactive
Omeprazol	Inactive	Inactive	Inactive	Inactive	Inactive	Inactive	Inactive	Inactive
Losartan	Inactive	Inactive	Inactive	Inactive	Inactive	Inactive	Inactive	Inactive

## Data Availability

Not Applicable.

## Supplementary Materials

The following supporting information can be downloaded at: https://www.mdpi.com/article/10.3390/molecules27227986/s1, Table S1: PPI subfamilies and compounds; Table S2: RF setup information; Table S3: LRG setup information; Table S4: SVM setup information; Table S5: RF metrics values; Table S6: LRF metrics values; Table S7: SVM metrics values; Table S8: Statistical values of RF models; Table S9: Statistical values of LRG models; Table S10: Statistical values of SVM models; Table S11: RF models validation results; Table S12: LRG models validation results; Table S13: SVM models validation results; Figure S1: RF metrics heatmap; Figure S2: LRG metrics heatmap; Figure S3: SVM metrics heatmap.

## Abbreviations

AI: artificial intelligence; ECFP, extended connectivity fingerprint; FN, False Negative; FP, False Positive; LRG, logistic regression; ML, machine learning; PPI, protein–protein interaction; RF, random forest; SVM, support vector machines; TN, true negative; TP, true positive.
