# Supplementary material for: Machine Learning Models to Predict Protein–Protein Interaction Inhibitors
Source: Molecules. 2022 Nov 17;27(22):7986. doi: 10.3390/molecules27227986 (PMC9694076; doi:10.3390/molecules27227986)
Supplement: Supplementary file 1 [file molecules-27-07986-s001.zip › molecules-2012841-supplementary.pdf]

molecules

SUPPLEMENTARY MATERIAL

Machine learning models to predict protein-protein interaction inhibitors

Bárbara I. Díaz-Eufracio, José L. Medina-Franco

DIFACQUIM research group, Department of Pharmacy, School of Chemistry, Universidad Nacional Autónoma de México, Avenida Universidad 3000, 04510 Mexico City, Mexico.

Contents

	Page
**Table S1.** PPI subfamilies and compounds.	S2
**Table S2.** RF setup information.	S3
**Table S3.** LRG setup information.	S6
**Table S4.** SVM setup information.	S9
**Table S5.** RF metrics values.	S11
**Table S6.** LRF metrics values.	S16
**Table S7.** SVM metrics values.	S20
**Table S8.** Statistical values of RF models.	S23
**Table S9.** Statistical values of LRG models.	S23
**Table S10.** Statistical values of SVM models.	S23
**Table S11.** RF models validation results.	S24
**Table S12.** LRG models validation results.	S25
**Table S13.** SVM models validation results.	S26
**Figure S1**. RF metrics heatmap.	S27
**Figure S2**. LRG metrics heatmap.	S28
**Figure S3**. SVM metrics heatmap.	S29

Table S1. PPI subfamilies and compounds.

**PPI Subfamily**	**Number of compounds**
BCL2-Like / BAX	326
Bromodomain / Histone	277
CD4 / gp120	119
CD80 / CD28	73
E2 / E1	10
FAK / VEGFR3	2
IL2 / IL2R	18
LEDGF / IN	74
LFA / ICAM	277
MDM2-Like / P53	551
MDM2-Like / P53, MDM2-like dimer	2
MDM2-like dimer	2
MENIN / MLL	20
MLLT1 / H3	19
Myc / Max	16
NRP / VEGF	6
PCNA trimer	10
Pygo PHD / H3	8
SETDB1 / H3	8
SPIN1 / H3	26
TNF trimer	1
TTR	50
UPAR / UPA	5
VEGF / VEGFR	4
VHL / HIF1α	16
WD40 / H3	7
WDR5/MLL	28
XIAP / Smac	272

Table S2. RF setup information.

FP	PK	Fingerprint	Test Set	Estimators	Criterion	Class weight
RF1	RFF1L6P3EN1A	ECFP4	0.2	100	entropy	balanced
RF2	RFF1L6P3EN1B	ECFP4	0.2	100	entropy	None
RF3	RFF1L6P3EN2A	ECFP4	0.2	500	entropy	balanced
RF4	RFF1L6P3EN2B	ECFP4	0.2	500	entropy	None
RF5	RFF1L6P3EN3A	ECFP4	0.2	1000	entropy	balanced
RF6	RFF1L6P3EN3B	ECFP4	0.2	1000	entropy	None
RF7	RFF1L6P3GN1A	ECFP4	0.2	100	gini	balanced
RF8	RFF1L6P3GN1B	ECFP4	0.2	100	gini	None
RF9	RFF1L6P3GN2A	ECFP4	0.2	500	gini	balanced
RF10	RFF1L6P3GN2B	ECFP4	0.2	500	gini	None
RF11	RFF1L6P3GN3A	ECFP4	0.2	1000	gini	balanced
RF12	RFF1L6P3GN3B	ECFP4	0.2	1000	gini	None
RF13	RFF1L6P5EN1A	ECFP4	0.3	100	entropy	balanced
RF14	RFF1L6P5EN1B	ECFP4	0.3	100	entropy	None
RF15	RFF1L6P5EN2A	ECFP4	0.3	500	entropy	balanced
RF16	RFF1L6P5EN2B	ECFP4	0.3	500	entropy	None
RF17	RFF1L6P5EN3A	ECFP4	0.3	1000	entropy	balanced
RF18	RFF1L6P5EN3B	ECFP4	0.3	1000	entropy	None
RF19	RFF1L6P5GN1A	ECFP4	0.3	100	gini	balanced
RF20	RFF1L6P5GN1B	ECFP4	0.3	100	gini	None
RF21	RFF1L6P5GN2A	ECFP4	0.3	500	gini	balanced
RF22	RFF1L6P5GN2B	ECFP4	0.3	500	gini	None
RF23	RFF1L6P5GN3A	ECFP4	0.3	1000	gini	balanced
RF24	RFF1L6P5GN3B	ECFP4	0.3	1000	gini	None
RF25	RFF2L6P3EN1A	ECFP6	0.2	100	entropy	balanced
RF26	RFF2L6P3EN1B	ECFP6	0.2	100	entropy	None
RF27	RFF2L6P3EN2A	ECFP6	0.2	500	entropy	balanced

RF28	RFF2L6P3EN2B	ECFP6	0.2	500	entropy	None
RF29	RFF2L6P3EN3A	ECFP6	0.2	1000	entropy	balanced
RF30	RFF2L6P3EN3B	ECFP6	0.2	1000	entropy	None
RF31	RFF2L6P3GN1A	ECFP6	0.2	100	gini	balanced
RF32	RFF2L6P3GN1B	ECFP6	0.2	100	gini	None
RF33	RFF2L6P3GN2A	ECFP6	0.2	500	gini	balanced
RF34	RFF2L6P3GN2B	ECFP6	0.2	500	gini	None
RF35	RFF2L6P3GN3A	ECFP6	0.2	1000	gini	balanced
RF36	RFF2L6P3GN3B	ECFP6	0.2	1000	gini	None
RF37	RFF2L6P5EN1A	ECFP6	0.3	100	entropy	balanced
RF38	RFF2L6P5EN1B	ECFP6	0.3	100	entropy	None
RF39	RFF2L6P5EN2A	ECFP6	0.3	500	entropy	balanced
RF40	RFF2L6P5EN2B	ECFP6	0.3	500	entropy	None
RF41	RFF2L6P5EN3A	ECFP6	0.3	1000	entropy	balanced
RF42	RFF2L6P5EN3B	ECFP6	0.3	1000	entropy	None
RF43	RFF2L6P5GN1A	ECFP6	0.3	100	gini	balanced
RF44	RFF2L6P5GN1B	ECFP6	0.3	100	gini	None
RF45	RFF2L6P5GN2A	ECFP6	0.3	500	gini	balanced
RF46	RFF2L6P5GN2B	ECFP6	0.3	500	gini	None
RF47	RFF2L6P5GN3A	ECFP6	0.3	1000	gini	balanced
RF48	RFF2L6P5GN3B	ECFP6	0.3	1000	gini	None
RF49	RFF3L6P3EN1A	MACCSKEYS	0.2	100	entropy	balanced
RF50	RFF3L6P3EN1B	MACCSKEYS	0.2	100	entropy	None
RF51	RFF3L6P3EN2A	MACCSKEYS	0.2	500	entropy	balanced
RF52	RFF3L6P3EN2B	MACCSKEYS	0.2	500	entropy	None
RF53	RFF3L6P3EN3A	MACCSKEYS	0.2	1000	entropy	balanced
RF54	RFF3L6P3EN3B	MACCSKEYS	0.2	1000	entropy	None
RF55	RFF3L6P3GN1A	MACCSKEYS	0.2	100	gini	balanced
RF56	RFF3L6P3GN1B	MACCSKEYS	0.2	100	gini	None
RF57	RFF3L6P3GN2A	MACCSKEYS	0.2	500	gini	balanced
RF58	RFF3L6P3GN2B	MACCSKEYS	0.2	500	gini	None
RF59	RFF3L6P3GN3A	MACCSKEYS	0.2	1000	gini	balanced

RF60	RFF3L6P3GN3B	MACCSKEYS	0.2	1000	gini	None
RF61	RFF3L6P5EN1A	MACCSKEYS	0.3	100	entropy	balanced
RF62	RFF3L6P5EN1B	MACCSKEYS	0.3	100	entropy	None
RF63	RFF3L6P5EN2A	MACCSKEYS	0.3	500	entropy	balanced
RF64	RFF3L6P5EN2B	MACCSKEYS	0.3	500	entropy	None
RF65	RFF3L6P5EN3A	MACCSKEYS	0.3	1000	entropy	balanced
RF66	RFF3L6P5EN3B	MACCSKEYS	0.3	1000	entropy	None
RF67	RFF3L6P5GN1A	MACCSKEYS	0.3	100	gini	balanced
RF68	RFF3L6P5GN1B	MACCSKEYS	0.3	100	gini	None
RF69	RFF3L6P5GN2A	MACCSKEYS	0.3	500	gini	balanced
RF70	RFF3L6P5GN2B	MACCSKEYS	0.3	500	gini	None
RF71	RFF3L6P5GN3A	MACCSKEYS	0.3	1000	gini	balanced
RF72	RFF3L6P5GN3B	MACCSKEYS	0.3	1000	gini	None
RF73	RFF4L6P3EN1A	AtomPairs	0.2	100	entropy	balanced
RF74	RFF4L6P3EN1B	AtomPairs	0.2	100	entropy	None
RF75	RFF4L6P3EN2A	AtomPairs	0.2	500	entropy	balanced
RF76	RFF4L6P3EN2B	AtomPairs	0.2	500	entropy	None
RF77	RFF4L6P3EN3A	AtomPairs	0.2	1000	entropy	balanced
RF78	RFF4L6P3EN3B	AtomPairs	0.2	1000	entropy	None
RF79	RFF4L6P3GN1A	AtomPairs	0.2	100	gini	balanced
RF80	RFF4L6P3GN1B	AtomPairs	0.2	100	gini	None
RF81	RFF4L6P3GN2A	AtomPairs	0.2	500	gini	balanced
RF82	RFF4L6P3GN2B	AtomPairs	0.2	500	gini	None
RF83	RFF4L6P3GN3A	AtomPairs	0.2	1000	gini	balanced
RF84	RFF4L6P3GN3B	AtomPairs	0.2	1000	gini	None
RF85	RFF4L6P5EN1A	AtomPairs	0.3	100	entropy	balanced
RF86	RFF4L6P5EN1B	AtomPairs	0.3	100	entropy	None
RF87	RFF4L6P5EN2A	AtomPairs	0.3	500	entropy	balanced
RF88	RFF4L6P5EN2B	AtomPairs	0.3	500	entropy	None
RF89	RFF4L6P5EN3A	AtomPairs	0.3	1000	entropy	balanced
RF90	RFF4L6P5EN3B	AtomPairs	0.3	1000	entropy	None
RF91	RFF4L6P5GN1A	AtomPairs	0.3	100	gini	balanced

RF92	RFF4L6P5GN1B	AtomPairs	0.3	100	gini	None
RF93	RFF4L6P5GN2A	AtomPairs	0.3	500	gini	balanced
RF94	RFF4L6P5GN2B	AtomPairs	0.3	500	gini	None
RF95	RFF4L6P5GN3A	AtomPairs	0.3	1000	gini	balanced
RF96	RFF4L6P5GN3B	AtomPairs	0.3	1000	gini	None

Table S3. LRG setup information.

**FK**	**PK**	**Fingerprint**	**Test set proportion**	**Solver**	**Class weight**
LRG1	LRGF1L6P3S1A	ECFP4	0.2	newton-cg	balanced
LRG2	LRGF1L6P3S1B	ECFP4	0.2	newton-cg	None
LRG3	LRGF1L6P3S2A	ECFP4	0.2	lbfgs	balanced
LRG4	LRGF1L6P3S2B	ECFP4	0.2	lbfgs	None
LRG5	LRGF1L6P3S3A	ECFP4	0.2	liblinear	balanced
LRG6	LRGF1L6P3S3B	ECFP4	0.2	liblinear	None
LRG7	LRGF1L6P3S4A	ECFP4	0.2	sag	balanced
LRG8	LRGF1L6P3S4B	ECFP4	0.2	sag	None
LRG9	LRGF1L6P3S5A	ECFP4	0.2	saga	balanced
LRG10	LRGF1L6P3S5B	ECFP4	0.2	saga	None
LRG11	LRGF1L6P5S1A	ECFP4	0.3	newton-cg	balanced
LRG12	LRGF1L6P5S1B	ECFP4	0.3	newton-cg	None
LRG13	LRGF1L6P5S2A	ECFP4	0.3	lbfgs	balanced
LRG14	LRGF1L6P5S2B	ECFP4	0.3	lbfgs	None
LRG15	LRGF1L6P5S3A	ECFP4	0.3	liblinear	balanced
LRG16	LRGF1L6P5S3B	ECFP4	0.3	liblinear	None
LRG17	LRGF1L6P5S4A	ECFP4	0.3	sag	balanced
LRG18	LRGF1L6P5S4B	ECFP4	0.3	sag	None
LRG19	LRGF1L6P5S5A	ECFP4	0.3	saga	balanced
LRG20	LRGF1L6P5S5B	ECFP4	0.3	saga	None
LRG21	LRGF2L6P3S1A	ECFP6	0.2	newton-cg	balanced
LRG22	LRGF2L6P3S1B	ECFP6	0.2	newton-cg	None
LRG23	LRGF2L6P3S2A	ECFP6	0.2	lbfgs	balanced

LRG24	LRGF2L6P3S2B	ECFP6	0.2	lbfgs	None
LRG25	LRGF2L6P3S3A	ECFP6	0.2	liblinear	balanced
LRG26	LRGF2L6P3S3B	ECFP6	0.2	liblinear	None
LRG27	LRGF2L6P3S4A	ECFP6	0.2	sag	balanced
LRG28	LRGF2L6P3S4B	ECFP6	0.2	sag	None
LRG29	LRGF2L6P3S5A	ECFP6	0.2	saga	balanced
LRG30	LRGF2L6P3S5B	ECFP6	0.2	saga	None
LRG31	LRGF2L6P5S1A	ECFP6	0.3	newton-cg	balanced
LRG32	LRGF2L6P5S1B	ECFP6	0.3	newton-cg	None
LRG33	LRGF2L6P5S2A	ECFP6	0.3	lbfgs	balanced
LRG34	LRGF2L6P5S2B	ECFP6	0.3	lbfgs	None
LRG35	LRGF2L6P5S3A	ECFP6	0.3	liblinear	balanced
LRG36	LRGF2L6P5S3B	ECFP6	0.3	liblinear	None
LRG37	LRGF2L6P5S4A	ECFP6	0.3	sag	balanced
LRG38	LRGF2L6P5S4B	ECFP6	0.3	sag	None
LRG39	LRGF2L6P5S5A	ECFP6	0.3	saga	balanced
LRG40	LRGF2L6P5S5B	ECFP6	0.3	saga	None
LRG41	LRGF3L6P3S1A	MACCSKEYS	0.2	newton-cg	balanced
LRG42	LRGF3L6P3S1B	MACCSKEYS	0.2	newton-cg	None
LRG43	LRGF3L6P3S2A	MACCSKEYS	0.2	lbfgs	balanced
LRG44	LRGF3L6P3S2B	MACCSKEYS	0.2	lbfgs	None
LRG45	LRGF3L6P3S3A	MACCSKEYS	0.2	liblinear	balanced
LRG46	LRGF3L6P3S3B	MACCSKEYS	0.2	liblinear	None
LRG47	LRGF3L6P3S4A	MACCSKEYS	0.2	sag	balanced
LRG48	LRGF3L6P3S4B	MACCSKEYS	0.2	sag	None
LRG49	LRGF3L6P3S5A	MACCSKEYS	0.2	saga	balanced
LRG50	LRGF3L6P3S5B	MACCSKEYS	0.2	saga	None
LRG51	LRGF3L6P5S1A	MACCSKEYS	0.3	newton-cg	balanced
LRG52	LRGF3L6P5S1B	MACCSKEYS	0.3	newton-cg	None
LRG53	LRGF3L6P5S2A	MACCSKEYS	0.3	lbfgs	balanced
LRG54	LRGF3L6P5S2B	MACCSKEYS	0.3	lbfgs	None
LRG55	LRGF3L6P5S3A	MACCSKEYS	0.3	liblinear	balanced

LRG56	LRGF3L6P5S3B	MACCSKEYS	0.3	liblinear	None
LRG57	LRGF3L6P5S4A	MACCSKEYS	0.3	sag	balanced
LRG58	LRGF3L6P5S4B	MACCSKEYS	0.3	sag	None
LRG59	LRGF3L6P5S5A	MACCSKEYS	0.3	saga	balanced
LRG60	LRGF3L6P5S5B	MACCSKEYS	0.3	saga	None
LRG61	LRGF4L6P3S1A	AtomPairs	0.2	newton-cg	balanced
LRG62	LRGF4L6P3S1B	AtomPairs	0.2	newton-cg	None
LRG63	LRGF4L6P3S2A	AtomPairs	0.2	lbfgs	balanced
LRG64	LRGF4L6P3S2B	AtomPairs	0.2	lbfgs	None
LRG65	LRGF4L6P3S3A	AtomPairs	0.2	liblinear	balanced
LRG66	LRGF4L6P3S3B	AtomPairs	0.2	liblinear	None
LRG67	LRGF4L6P3S4A	AtomPairs	0.2	sag	balanced
LRG68	LRGF4L6P3S4B	AtomPairs	0.2	sag	None
LRG69	LRGF4L6P3S5A	AtomPairs	0.2	saga	balanced
LRG70	LRGF4L6P3S5B	AtomPairs	0.2	saga	None
LRG71	LRGF4L6P5S1A	AtomPairs	0.3	newton-cg	balanced
LRG72	LRGF4L6P5S1B	AtomPairs	0.3	newton-cg	None
LRG73	LRGF4L6P5S2A	AtomPairs	0.3	lbfgs	balanced
LRG74	LRGF4L6P5S2B	AtomPairs	0.3	lbfgs	None
LRG75	LRGF4L6P5S3A	AtomPairs	0.3	liblinear	balanced
LRG76	LRGF4L6P5S3B	AtomPairs	0.3	liblinear	None
LRG77	LRGF4L6P5S4A	AtomPairs	0.3	sag	balanced
LRG78	LRGF4L6P5S4B	AtomPairs	0.3	sag	None
LRG79	LRGF4L6P5S5A	AtomPairs	0.3	saga	balanced
LRG80	LRGF4L6P5S5B	AtomPairs	0.3	saga	None

Table S4. SVM setup information.

**FK**	**PK**	**Fingerprint**	**Test set proportion**	**Kernel**	**Class weight**
SVM1	SVMF1L6P3K1A	ECFP4	0.2	linear	Balanced
SVM2	SVMF1L6P3K1B	ECFP4	0.2	linear	None
SVM3	SVMF1L6P3K2A	ECFP4	0.2	poly	Balanced

SVM4	SVMF1L6P3K2B	ECFP4	0.2	poly	None
SVM5	SVMF1L6P3K3A	ECFP4	0.2	rbf	Balanced
SVM6	SVMF1L6P3K3B	ECFP4	0.2	rbf	None
SVM7	SVMF1L6P3K4A	ECFP4	0.2	sigmoid	Balanced
SVM8	SVMF1L6P3K4B	ECFP4	0.2	sigmoid	None
SVM9	SVMF1L6P5K1A	ECFP4	0.3	linear	Balanced
SVM10	SVMF1L6P5K1B	ECFP4	0.3	linear	None
SVM11	SVMF1L6P5K2A	ECFP4	0.3	poly	Balanced
SVM12	SVMF1L6P5K2B	ECFP4	0.3	poly	None
SVM13	SVMF1L6P5K3A	ECFP4	0.3	rbf	Balanced
SVM14	SVMF1L6P5K3B	ECFP4	0.3	rbf	None
SVM15	SVMF1L6P5K4A	ECFP4	0.3	sigmoid	Balanced
SVM16	SVMF1L6P5K4B	ECFP4	0.3	sigmoid	None
SVM17	SVMF2L6P3K1A	ECFP6	0.2	linear	Balanced
SVM18	SVMF2L6P3K1B	ECFP6	0.2	linear	None
SVM19	SVMF2L6P3K2A	ECFP6	0.2	poly	Balanced
SVM20	SVMF2L6P3K2B	ECFP6	0.2	poly	None
SVM21	SVMF2L6P3K3A	ECFP6	0.2	rbf	Balanced
SVM22	SVMF2L6P3K3B	ECFP6	0.2	rbf	None
SVM23	SVMF2L6P3K4A	ECFP6	0.2	sigmoid	Balanced
SVM24	SVMF2L6P3K4B	ECFP6	0.2	sigmoid	None
SVM25	SVMF2L6P5K1A	ECFP6	0.3	linear	Balanced
SVM26	SVMF2L6P5K1B	ECFP6	0.3	linear	None
SVM27	SVMF2L6P5K2A	ECFP6	0.3	poly	Balanced
SVM28	SVMF2L6P5K2B	ECFP6	0.3	poly	None
SVM29	SVMF2L6P5K3A	ECFP6	0.3	rbf	Balanced
SVM30	SVMF2L6P5K3B	ECFP6	0.3	rbf	None
SVM31	SVMF2L6P5K4A	ECFP6	0.3	sigmoid	Balanced
SVM32	SVMF2L6P5K4B	ECFP6	0.3	sigmoid	None
SVM33	SVMF3L6P3K1A	MACCSKEYS	0.2	linear	Balanced
SVM34	SVMF3L6P3K1B	MACCSKEYS	0.2	linear	None
SVM35	SVMF3L6P3K2A	MACCSKEYS	0.2	poly	Balanced

SVM36	SVMF3L6P3K2B	MACCSKEYS	0.2	poly	None
SVM37	SVMF3L6P3K3A	MACCSKEYS	0.2	rbf	Balanced
SVM38	SVMF3L6P3K3B	MACCSKEYS	0.2	rbf	None
SVM39	SVMF3L6P3K4A	MACCSKEYS	0.2	sigmoid	Balanced
SVM40	SVMF3L6P3K4B	MACCSKEYS	0.2	sigmoid	None
SVM41	SVMF3L6P5K1A	MACCSKEYS	0.3	linear	Balanced
SVM42	SVMF3L6P5K1B	MACCSKEYS	0.3	linear	None
SVM43	SVMF3L6P5K2A	MACCSKEYS	0.3	poly	Balanced
SVM44	SVMF3L6P5K2B	MACCSKEYS	0.3	poly	None
SVM45	SVMF3L6P5K3A	MACCSKEYS	0.3	rbf	Balanced
SVM46	SVMF3L6P5K3B	MACCSKEYS	0.3	rbf	None
SVM47	SVMF3L6P5K4A	MACCSKEYS	0.3	sigmoid	Balanced
SVM48	SVMF3L6P5K4B	MACCSKEYS	0.3	sigmoid	None
SVM49	SVMF4L6P3K1A	AtomPairs	0.2	linear	Balanced
SVM50	SVMF4L6P3K1B	AtomPairs	0.2	linear	None
SVM51	SVMF4L6P3K2A	AtomPairs	0.2	poly	Balanced
SVM52	SVMF4L6P3K2B	AtomPairs	0.2	poly	None
SVM53	SVMF4L6P3K3A	AtomPairs	0.2	rbf	Balanced
SVM54	SVMF4L6P3K3B	AtomPairs	0.2	rbf	None
SVM55	SVMF4L6P3K4A	AtomPairs	0.2	sigmoid	Balanced
SVM56	SVMF4L6P3K4B	AtomPairs	0.2	sigmoid	None
SVM57	SVMF4L6P5K1A	AtomPairs	0.3	linear	Balanced
SVM58	SVMF4L6P5K1B	AtomPairs	0.3	linear	None
SVM59	SVMF4L6P5K2A	AtomPairs	0.3	poly	Balanced
SVM60	SVMF4L6P5K2B	AtomPairs	0.3	poly	None
SVM61	SVMF4L6P5K3A	AtomPairs	0.3	rbf	Balanced
SVM62	SVMF4L6P5K3B	AtomPairs	0.3	rbf	None
SVM63	SVMF4L6P5K4A	AtomPairs	0.3	sigmoid	Balanced
SVM64	SVMF4L6P5K4B	AtomPairs	0.3	sigmoid	None

Table S5. RF metrics values.

	Accuracy	Balanced Accuracy	Precision	F1	Recall	Confusion matrix
RF1	0.95	0.95	0.98	0.95	0.92	[483 10]<br>[33 400]
RF2	0.95	0.95	0.97	0.95	0.93	[480 13]<br>[29 404]
RF3	0.96	0.96	0.97	0.95	0.94	[481 12]<br>[27 406]
RF4	0.96	0.96	0.98	0.96	0.94	[483 10]<br>[28 405]
RF5	0.95	0.95	0.97	0.95	0.93	[481 12]<br>[30 403]
RF6	0.96	0.96	0.98	0.96	0.94	[483 10]<br>[28 405]
RF7	0.96	0.96	0.97	0.95	0.94	[480 13]<br>[26 407]
RF8	0.95	0.95	0.96	0.95	0.94	[477 16]<br>[26 407]
RF9	0.96	0.96	0.97	0.95	0.94	[480 13]<br>[26 407]
RF10	0.96	0.95	0.96	0.95	0.94	[478 15]<br>[26 407]
RF11	0.96	0.96	0.97	0.95	0.94	[480 13]<br>[27 406]
RF12	0.96	0.95	0.97	0.95	0.94	[479 14]<br>[27 406]
RF13	0.95	0.95	0.96	0.95	0.94	[711 25]<br>[40 613]
RF14	0.95	0.95	0.95	0.95	0.94	[704 32]<br>[38 615]
RF15	0.95	0.95	0.96	0.95	0.94	[711 25]<br>[41 612]
RF16	0.95	0.95	0.96	0.95	0.94	[710 26]<br>[41 612]
RF17	0.95	0.95	0.96	0.95	0.94	[712 24]<br>[41 612]
RF18	0.95	0.95	0.96	0.95	0.94	[709 27]<br>[42 611]
RF19	0.95	0.95	0.96	0.95	0.94	[709 27]<br>[39 614]

RF20	0.95	0.95	0.95	0.95	0.95	[705 31]<br>[33 620]
RF21	0.96	0.95	0.96	0.95	0.95	[708 28]<br>[34 619]
RF22	0.95	0.95	0.96	0.95	0.94	[709 27]<br>[38 615]
RF23	0.95	0.95	0.96	0.95	0.94	[709 27]<br>[37 616]
RF24	0.95	0.95	0.96	0.95	0.94	[709 27]<br>[36 617]
RF25	0.95	0.95	0.98	0.95	0.92	[483 10]<br>[33 400]
RF26	0.95	0.95	0.98	0.95	0.91	[484 9]<br>[37 396]
RF27	0.96	0.96	0.98	0.95	0.93	[486 7]<br>[32 401]
RF28	0.95	0.95	0.98	0.95	0.92	[484 9]<br>[35 398]
RF29	0.95	0.95	0.98	0.95	0.92	[483 10]<br>[35 398]
RF30	0.95	0.95	0.97	0.95	0.92	[482 11]<br>[35 398]
RF31	0.95	0.95	0.96	0.94	0.92	[477 16]<br>[33 400]
RF32	0.95	0.95	0.97	0.95	0.93	[479 14]<br>[29 404]
RF33	0.95	0.95	0.97	0.95	0.93	[481 12]<br>[32 401]
RF34	0.95	0.95	0.97	0.95	0.93	[479 14]<br>[30 403]
RF35	0.95	0.95	0.97	0.95	0.93	[481 12]<br>[30 403]
RF36	0.95	0.95	0.97	0.95	0.93	[479 14]<br>[29 404]
RF37	0.95	0.95	0.96	0.94	0.93	[709 27]<br>[47 606]
RF38	0.95	0.95	0.96	0.95	0.93	[711 25]<br>[45 608]
RF39	0.95	0.95	0.96	0.95	0.93	[710 26]<br>[44 609]

RF40	0.95	0.95	0.96	0.95	0.93	[711 25]<br>[43 610]
RF41	0.95	0.95	0.96	0.95	0.93	[711 25]<br>[45 608]
RF42	0.95	0.95	0.96	0.95	0.93	[712 24]<br>[43 610]
RF43	0.95	0.95	0.95	0.94	0.93	[706 30]<br>[43 610]
RF44	0.95	0.95	0.95	0.94	0.93	[706 30]<br>[45 608]
RF45	0.95	0.95	0.96	0.95	0.93	[711 25]<br>[43 610]
RF46	0.95	0.94	0.95	0.94	0.93	[706 30]<br>[46 607]
RF47	0.95	0.95	0.96	0.94	0.93	[709 27]<br>[45 608]
RF48	0.94	0.94	0.95	0.94	0.93	[703 33]<br>[45 608]
RF49	0.94	0.94	0.93	0.93	0.94	[463 30]<br>[28 405]
RF50	0.94	0.94	0.93	0.94	0.94	[462 31]<br>[25 408]
RF51	0.94	0.94	0.93	0.94	0.94	[463 30]<br>[25 408]
RF52	0.94	0.94	0.93	0.94	0.95	[462 31]<br>[23 410]
RF53	0.94	0.94	0.93	0.94	0.94	[462 31]<br>[25 408]
RF54	0.94	0.94	0.93	0.94	0.95	[463 30]<br>[23 410]
RF55	0.94	0.94	0.93	0.93	0.94	[463 30]<br>[27 406]
RF56	0.94	0.94	0.92	0.93	0.94	[460 33]<br>[27 406]
RF57	0.94	0.94	0.93	0.94	0.94	[462 31]<br>[24 409]
RF58	0.94	0.94	0.93	0.93	0.94	[460 33]<br>[24 409]
RF59	0.94	0.94	0.93	0.94	0.94	[462 31]<br>[24 409]

RF60	0.94	0.94	0.93	0.94	0.94	[461 32]<br>[24 409]
RF61	0.93	0.93	0.91	0.93	0.94	[676 60]<br>[36 617]
RF62	0.93	0.93	0.91	0.93	0.95	[676 60]<br>[32 621]
RF63	0.93	0.93	0.91	0.93	0.95	[676 60]<br>[33 620]
RF64	0.93	0.93	0.91	0.93	0.95	[676 60]<br>[32 621]
RF65	0.93	0.93	0.91	0.93	0.95	[674 62]<br>[31 622]
RF66	0.94	0.94	0.91	0.93	0.95	[677 59]<br>[31 622]
RF67	0.93	0.93	0.92	0.93	0.95	[679 57]<br>[35 618]
RF68	0.94	0.94	0.92	0.94	0.95	[684 52]<br>[33 620]
RF69	0.94	0.94	0.92	0.93	0.95	[679 57]<br>[31 622]
RF70	0.94	0.94	0.92	0.94	0.96	[685 51]<br>[29 624]
RF71	0.94	0.94	0.92	0.94	0.95	[681 55]<br>[31 622]
RF72	0.94	0.94	0.92	0.94	0.96	[682 54]<br>[29 624]
RF73	0.94	0.94	0.93	0.93	0.94	[461 32]<br>[27 406]
RF74	0.93	0.93	0.92	0.93	0.94	[456 37]<br>[27 406]
RF75	0.93	0.93	0.92	0.93	0.94	[456 37]<br>[27 406]
RF76	0.93	0.93	0.92	0.93	0.94	[457 36]<br>[27 406]
RF77	0.93	0.93	0.91	0.93	0.94	[454 39]<br>[26 407]
RF78	0.93	0.93	0.92	0.93	0.94	[456 37]<br>[27 406]
RF79	0.94	0.94	0.93	0.93	0.94	[461 32]<br>[28 405]

RF80	0.93	0.93	0.92	0.93	0.94	[457 36]<br>[27 406]
RF81	0.93	0.93	0.92	0.93	0.94	[456 37]<br>[27 406]
RF82	0.94	0.94	0.92	0.93	0.94	[458 35]<br>[24 409]
RF83	0.93	0.93	0.92	0.93	0.94	[456 37]<br>[26 407]
RF84	0.93	0.93	0.91	0.93	0.94	[455 38]<br>[25 408]
RF85	0.94	0.94	0.93	0.93	0.94	[689 47]<br>[42 611]
RF86	0.93	0.93	0.93	0.93	0.94	[687 49]<br>[42 611]
RF87	0.93	0.93	0.92	0.93	0.94	[684 52]<br>[42 611]
RF88	0.93	0.93	0.92	0.93	0.94	[683 53]<br>[40 613]
RF89	0.93	0.93	0.92	0.93	0.94	[684 52]<br>[41 612]
RF90	0.93	0.93	0.92	0.93	0.94	[682 54]<br>[40 613]
RF91	0.93	0.93	0.92	0.93	0.94	[684 52]<br>[41 612]
RF92	0.93	0.93	0.92	0.93	0.94	[682 54]<br>[39 614]
RF93	0.93	0.93	0.92	0.93	0.94	[683 53]<br>[39 614]
RF94	0.93	0.93	0.92	0.93	0.94	[680 56]<br>[41 612]
RF95	0.93	0.93	0.92	0.93	0.94	[683 53]<br>[41 612]
RF96	0.93	0.93	0.91	0.93	0.94	[678 58]<br>[39 614]

Table S6. LRF metrics values.

	**Accuracy**	**Balanced Accuracy**	**Precision**	**F1**	**Recall**	**Confusion matrix**
LRG1	0.94	0.94	0.94	0.94	0.93	[469 24]<br>[30 403]

LRG2	0.94	0.94	0.94	0.94	0.93	[469 24]<br>[30 403]
LRG3	0.94	0.94	0.94	0.94	0.93	[469 24]<br>[30 403]
LRG4	0.94	0.94	0.94	0.94	0.93	[469 24]<br>[30 403]
LRG5	0.94	0.94	0.94	0.93	0.93	[467 26]<br>[31 402]
LRG6	0.94	0.94	0.94	0.93	0.93	[467 26]<br>[32 401]
LRG7	0.94	0.94	0.94	0.94	0.93	[469 24]<br>[30 403]
LRG8	0.94	0.94	0.94	0.94	0.93	[469 24]<br>[30 403]
LRG9	0.94	0.94	0.94	0.94	0.93	[468 25]<br>[30 403]
LRG10	0.94	0.94	0.95	0.94	0.93	[471 22]<br>[31 402]
LRG11	0.94	0.94	0.93	0.94	0.94	[689 47]<br>[36 617]
LRG12	0.94	0.94	0.93	0.94	0.94	[689 47]<br>[36 617]
LRG13	0.94	0.94	0.93	0.94	0.94	[689 47]<br>[36 617]
LRG14	0.94	0.94	0.93	0.94	0.94	[689 47]<br>[36 617]
LRG15	0.94	0.94	0.93	0.93	0.94	[688 48]<br>[38 615]
LRG16	0.94	0.94	0.93	0.93	0.94	[688 48]<br>[38 615]
LRG17	0.94	0.94	0.93	0.94	0.94	[689 47]<br>[36 617]
LRG18	0.94	0.94	0.93	0.94	0.94	[689 47]<br>[37 616]
LRG19	0.94	0.94	0.93	0.94	0.94	[688 48]<br>[37 616]
LRG20	0.94	0.94	0.93	0.94	0.94	[688 48]<br>[37 616]
LRG21	0.95	0.95	0.94	0.94	0.94	[469 24]<br>[26 407]

LRG22	0.95	0.95	0.95	0.94	0.94	[470 23]<br>[27 406]
LRG23	0.95	0.95	0.94	0.94	0.94	[469 24]<br>[26 407]
LRG24	0.95	0.95	0.95	0.94	0.94	[470 23]<br>[27 406]
LRG25	0.94	0.94	0.94	0.94	0.94	[465 28]<br>[27 406]
LRG26	0.94	0.94	0.94	0.94	0.94	[465 28]<br>[28 405]
LRG27	0.95	0.95	0.95	0.94	0.94	[470 23]<br>[26 407]
LRG28	0.94	0.94	0.95	0.94	0.94	[470 23]<br>[28 405]
LRG29	0.94	0.94	0.94	0.94	0.94	[469 24]<br>[27 406]
LRG30	0.94	0.94	0.95	0.94	0.94	[470 23]<br>[28 405]
LRG31	0.94	0.94	0.93	0.94	0.94	[687 49]<br>[36 617]
LRG32	0.94	0.94	0.93	0.93	0.94	[687 49]<br>[38 615]
LRG33	0.94	0.94	0.93	0.94	0.94	[687 49]<br>[36 617]
LRG34	0.94	0.94	0.93	0.93	0.94	[687 49]<br>[38 615]
LRG35	0.94	0.94	0.93	0.94	0.95	[690 46]<br>[33 620]
LRG36	0.94	0.94	0.93	0.94	0.95	[690 46]<br>[33 620]
LRG37	0.94	0.94	0.92	0.93	0.94	[685 51]<br>[36 617]
LRG38	0.94	0.94	0.93	0.94	0.94	[687 49]<br>[36 617]
LRG39	0.94	0.94	0.93	0.94	0.95	[687 49]<br>[33 620]
LRG40	0.94	0.94	0.93	0.94	0.95	[688 48]<br>[34 619]
LRG41	0.86	0.86	0.84	0.86	0.88	[421 72]<br>[54 379]

LRG42	0.86	0.86	0.84	0.86	0.87	[421 72]<br>[55 378]
LRG43	0.86	0.86	0.84	0.86	0.88	[421 72]<br>[54 379]
LRG44	0.86	0.86	0.84	0.86	0.87	[421 72]<br>[55 378]
LRG45	0.87	0.87	0.84	0.86	0.88	[422 71]<br>[54 379]
LRG46	0.87	0.87	0.84	0.86	0.88	[422 71]<br>[54 379]
LRG47	0.86	0.86	0.84	0.86	0.88	[421 72]<br>[54 379]
LRG48	0.86	0.86	0.84	0.86	0.88	[421 72]<br>[54 379]
LRG49	0.86	0.86	0.84	0.86	0.88	[421 72]<br>[54 379]
LRG50	0.86	0.86	0.84	0.86	0.88	[421 72]<br>[54 379]
LRG51	0.87	0.87	0.84	0.86	0.88	[627 109]<br>[78 575]
LRG52	0.86	0.86	0.84	0.86	0.87	[629 107]<br>[83 570]
LRG53	0.87	0.87	0.84	0.86	0.88	[627 109]<br>[77 576]
LRG54	0.86	0.86	0.84	0.86	0.87	[630 106]<br>[84 569]
LRG55	0.87	0.87	0.84	0.86	0.88	[626 110]<br>[77 576]
LRG56	0.87	0.87	0.85	0.86	0.87	[633 103]<br>[82 571]
LRG57	0.87	0.87	0.84	0.86	0.88	[627 109]<br>[78 575]
LRG58	0.86	0.86	0.84	0.86	0.87	[629 107]<br>[83 570]
LRG59	0.87	0.87	0.84	0.86	0.88	[627 109]<br>[77 576]
LRG60	0.86	0.86	0.84	0.86	0.87	[630 106]<br>[83 570]
LRG61	0.94	0.94	0.93	0.93	0.94	[461 32]<br>[28 405]

LRG62	0.93	0.93	0.93	0.93	0.93	[461 32]<br>[29 404]
LRG63	0.94	0.94	0.93	0.93	0.94	[461 32]<br>[26 407]
LRG64	0.94	0.94	0.92	0.93	0.94	[460 33]<br>[27 406]
LRG65	0.94	0.94	0.93	0.93	0.94	[461 32]<br>[27 406]
LRG66	0.93	0.93	0.92	0.93	0.94	[459 34]<br>[27 406]
LRG67	0.84	0.85	0.81	0.84	0.87	[407 86]<br>[58 375]
LRG68	0.85	0.85	0.82	0.84	0.86	[412 81]<br>[62 371]
LRG69	0.84	0.84	0.78	0.84	0.9	[384 109]<br>[43 390]
LRG70	0.83	0.84	0.79	0.83	0.89	[389 104]<br>[49 384]
LRG71	0.93	0.93	0.91	0.92	0.94	[676 60]<br>[42 611]
LRG72	0.93	0.93	0.91	0.92	0.93	[677 59]<br>[43 610]
LRG73	0.93	0.93	0.91	0.92	0.93	[679 57]<br>[44 609]
LRG74	0.93	0.93	0.91	0.92	0.94	[678 58]<br>[42 611]
LRG75	0.93	0.93	0.91	0.92	0.94	[676 60]<br>[41 612]
LRG76	0.93	0.93	0.91	0.92	0.94	[675 61]<br>[42 611]
LRG77	0.85	0.85	0.81	0.84	0.89	[596 140]<br>[74 579]
LRG78	0.85	0.85	0.81	0.84	0.88	[603 133]<br>[80 573]
LRG79	0.83	0.83	0.76	0.83	0.91	[550 186]<br>[56 597]
LRG80	0.83	0.83	0.77	0.83	0.91	[557 179]<br>[61 592]

Table S7. SVM metrics values.

	**Accuracy**	**Balanced Accuracy**	**Precision**	**F1**	**Recall**	**Confusion matrix**
SVM1	0.94	0.94	0.94	0.94	0.94	[465 28]<br>[27 406]
SVM2	0.94	0.94	0.93	0.93	0.94	[463 30]<br>[27 406]
SVM3	0.94	0.94	0.98	0.93	0.89	[487 6]<br>[49 384]
SVM4	0.94	0.94	0.98	0.93	0.89	[487 6]<br>[49 384]
SVM5	0.95	0.95	0.97	0.95	0.93	[481 12]<br>[31 402]
SVM6	0.95	0.95	0.97	0.95	0.92	[482 11]<br>[33 400]
SVM7	0.9	0.9	0.89	0.9	0.9	[446 47]<br>[43 390]
SVM8	0.9	0.9	0.89	0.9	0.9	[446 47]<br>[43 390]
SVM9	0.94	0.94	0.93	0.94	0.94	[689 47]<br>[38 615]
SVM10	0.94	0.94	0.93	0.94	0.94	[690 46]<br>[38 615]
SVM11	0.94	0.94	0.98	0.93	0.89	[724 12]<br>[73 580]
SVM12	0.94	0.93	0.98	0.93	0.89	[724 12]<br>[75 578]
SVM13	0.95	0.95	0.96	0.95	0.94	[713 23]<br>[42 611]
SVM14	0.95	0.95	0.96	0.95	0.93	[713 23]<br>[43 610]
SVM15	0.91	0.91	0.89	0.9	0.91	[664 72]<br>[57 596]
SVM16	0.91	0.91	0.9	0.9	0.91	[668 68]<br>[62 591]
SVM17	0.94	0.94	0.93	0.93	0.93	[464 29]<br>[29 404]
SVM18	0.94	0.93	0.93	0.93	0.93	[465 28]<br>[32 401]
SVM19	0.93	0.92	0.99	0.92	0.86	[488 5]<br>[61 372]

SVM20	0.93	0.92	0.99	0.92	0.86	[488 5]<br>[62 371]
SVM21	0.95	0.95	0.98	0.95	0.92	[483 10]<br>[33 400]
SVM22	0.95	0.95	0.98	0.95	0.92	[484 9]<br>[33 400]
SVM23	0.92	0.92	0.91	0.91	0.91	[453 40]<br>[37 396]
SVM24	0.92	0.92	0.91	0.91	0.91	[455 38]<br>[40 393]
SVM25	0.93	0.93	0.91	0.93	0.94	[677 59]<br>[36 617]
SVM26	0.93	0.93	0.92	0.93	0.94	[679 57]<br>[38 615]
SVM27	0.93	0.92	0.98	0.92	0.87	[722 14]<br>[88 565]
SVM28	0.93	0.92	0.98	0.92	0.86	[722 14]<br>[89 564]
SVM29	0.95	0.95	0.97	0.95	0.93	[714 22]<br>[46 607]
SVM30	0.95	0.95	0.97	0.95	0.93	[714 22]<br>[46 607]
SVM31	0.91	0.91	0.9	0.91	0.91	[668 68]<br>[56 597]
SVM32	0.91	0.91	0.9	0.9	0.91	[669 67]<br>[60 593]
SVM33	0.87	0.88	0.85	0.87	0.88	[427 66]<br>[50 383]
SVM34	0.87	0.87	0.85	0.86	0.87	[428 65]<br>[56 377]
SVM35	0.94	0.94	0.94	0.93	0.93	[465 28]<br>[30 403]
SVM36	0.94	0.94	0.94	0.93	0.93	[466 27]<br>[32 401]
SVM37	0.92	0.92	0.91	0.92	0.93	[451 42]<br>[29 404]
SVM38	0.92	0.92	0.91	0.92	0.93	[452 41]<br>[32 401]
SVM39	0.62	0.62	0.59	0.59	0.58	[322 171]<br>[182 251]

SVM40	0.62	0.62	0.6	0.58	0.57	[327 166]<br>[186 247]
SVM41	0.88	0.88	0.85	0.87	0.9	[632 104]<br>[66 587]
SVM42	0.88	0.88	0.85	0.87	0.89	[637 99]<br>[71 582]
SVM43	0.94	0.94	0.93	0.93	0.93	[690 46]<br>[43 610]
SVM44	0.94	0.94	0.93	0.93	0.93	[691 45]<br>[43 610]
SVM45	0.92	0.92	0.9	0.92	0.94	[667 69]<br>[41 612]
SVM46	0.92	0.92	0.9	0.92	0.94	[668 68]<br>[42 611]
SVM47	0.62	0.62	0.6	0.6	0.6	[473 263]<br>[260 393]
SVM48	0.63	0.63	0.61	0.6	0.59	[485 251]<br>[265 388]
SVM49	0.93	0.93	0.91	0.92	0.93	[455 38]<br>[30 403]
SVM50	0.92	0.92	0.91	0.92	0.93	[452 41]<br>[32 401]
SVM51	0.81	0.8	0.91	0.77	0.66	[464 29]<br>[146 287]
SVM52	0.81	0.8	0.91	0.76	0.66	[464 29]<br>[148 285]
SVM53	0.89	0.89	0.9	0.88	0.86	[451 42]<br>[59 374]
SVM54	0.89	0.89	0.9	0.88	0.86	[454 39]<br>[62 371]
SVM55	0.79	0.79	0.79	0.77	0.75	[407 86]<br>[109 324]
SVM56	0.79	0.78	0.8	0.76	0.73	[411 82]<br>[115 318]
SVM57	0.91	0.91	0.89	0.91	0.93	[659 77]<br>[47 606]
SVM58	0.91	0.91	0.89	0.91	0.93	[662 74]<br>[47 606]
SVM59	0.82	0.81	0.91	0.78	0.69	[690 46]<br>[205 448]

SVM60	0.82	0.81	0.91	0.78	0.68	[691 45]<br>[208 445]
SVM61	0.89	0.89	0.89	0.88	0.87	[669 67]<br>[84 569]
SVM62	0.89	0.89	0.9	0.88	0.87	[670 66]<br>[85 568]
SVM63	0.81	0.8	0.81	0.79	0.77	[614 122]<br>[147 506]
SVM64	0.81	0.8	0.81	0.79	0.77	[614 122]<br>[148 505]

Table S8. Statistical values of RF models.

	**count**	**mean**	**std**	**min**	**25%**	**50%**	**75%**	**max**
Accuracy	96	0.94	0.01	0.93	0.93	0.94	0.95	0.96
Balanced Accuracy	96	0.94	0.01	0.93	0.93	0.94	0.95	0.96
Precision	96	0.94	0.02	0.91	0.92	0.94	0.96	0.98
F1	96	0.94	0.01	0.93	0.93	0.94	0.95	0.96
Recall	96	0.94	0.01	0.91	0.93	0.94	0.94	0.96

Table S9. Statistical values of LRG models.

	**count**	**mean**	**std**	**min**	**25%**	**50%**	**75%**	**max**
Accuracy	80	0.91	0.04	0.83	0.87	0.94	0.94	0.95
Balanced Accuracy	80	0.91	0.04	0.83	0.87	0.94	0.94	0.95
Precision	80	0.9	0.05	0.76	0.84	0.93	0.93	0.95
F1	80	0.91	0.04	0.83	0.86	0.93	0.94	0.94
Recall	80	0.92	0.03	0.86	0.88	0.93	0.94	0.95

Table S10. Statistical values of SVM models.

	**count**	**mean**	**std**	**min**	**25%**	**50%**	**75%**	**max**
Accuracy	64	0.89	0.08	0.62	0.89	0.92	0.94	0.95
Balanced Accuracy	64	0.89	0.08	0.62	0.89	0.92	0.94	0.95

Precision	64	0.90	0.09	0.59	0.89	0.91	0.94	0.99
F1	64	0.88	0.09	0.58	0.88	0.92	0.93	0.95
Recall	64	0.87	0.1	0.57	0.86	0.91	0.93	0.94

Table S11. RF models validation results.

model	accuracy mean	accuracy std
RF3	0.957	0.013
RF4	0.958	0.014
RF6	0.958	0.014
RF7	0.959	0.012
RF8	0.955	0.015
RF9	0.957	0.013
RF10	0.956	0.014
RF11	0.957	0.013
RF12	0.957	0.013
RF13	0.958	0.014
RF14	0.958	0.012
RF15	0.957	0.013
RF16	0.958	0.014
RF17	0.957	0.013
RF18	0.958	0.014
RF19	0.959	0.012
RF20	0.955	0.015
RF21	0.957	0.013
RF22	0.956	0.014
RF23	0.957	0.013
RF24	0.957	0.013
RF27	0.957	0.014

Note: only those models with metrics greater than the statistical metric Q2 were validated.

Table S12. LRG models validation results.

Model	accuracy mean	accuracy std
LRG1	0.948	0.017

LRG2	0.949	0.017
LRG3	0.948	0.017
LRG4	0.949	0.017
LRG5	0.949	0.017
LRG6	0.949	0.017
LRG7	0.948	0.017
LRG8	0.949	0.017
LRG9	0.95	0.016
LRG10	0.95	0.017
LRG11	0.948	0.017
LRG12	0.949	0.017
LRG13	0.948	0.017
LRG14	0.949	0.017
LRG15	0.949	0.017
LRG16	0.949	0.017
LRG17	0.948	0.017
LRG18	0.949	0.017
LRG19	0.95	0.016
LRG20	0.95	0.017
LRG21	0.941	0.017
LRG22	0.941	0.017
LRG23	0.941	0.017
LRG24	0.941	0.017
LRG25	0.941	0.017
LRG26	0.94	0.018
LRG27	0.941	0.017
LRG28	0.941	0.018
LRG29	0.942	0.017
LRG30	0.942	0.017
LRG31	0.941	0.017
LRG32	0.941	0.017
LRG33	0.941	0.017
LRG34	0.941	0.017
LRG35	0.941	0.017

LRG36	0.94	0.018
LRG38	0.941	0.018
LRG39	0.942	0.017
LRG40	0.942	0.017
LRG61	0.938	0.014
LRG63	0.938	0.015
LRG65	0.937	0.015

Note: only those models with metrics greater than the statistical metric Q2 were validated.

Table S13. SVM models validation results.

**Model**	**accuracy mean**	**accuracy std**
SVM1	0.941	0.014
SVM2	0.942	0.014
SVM5	0.963	0.014
SVM6	0.963	0.013
SVM9	0.941	0.014
SVM10	0.942	0.014
SVM13	0.963	0.014
SVM14	0.963	0.013
SVM17	0.935	0.021
SVM18	0.937	0.019
SVM21	0.958	0.015
SVM22	0.958	0.015
SVM25	0.935	0.021
SVM26	0.937	0.019
SVM29	0.958	0.015
SVM30	0.958	0.015
SVM35	0.94	0.015
SVM36	0.939	0.015
SVM37	0.926	0.016
SVM38	0.927	0.017
SVM43	0.94	0.015
SVM44	0.939	0.015
SVM49	0.914	0.017

SVM50	0.916	0.017

Note: only those models with metrics greater than the statistical metric Q2 were validated

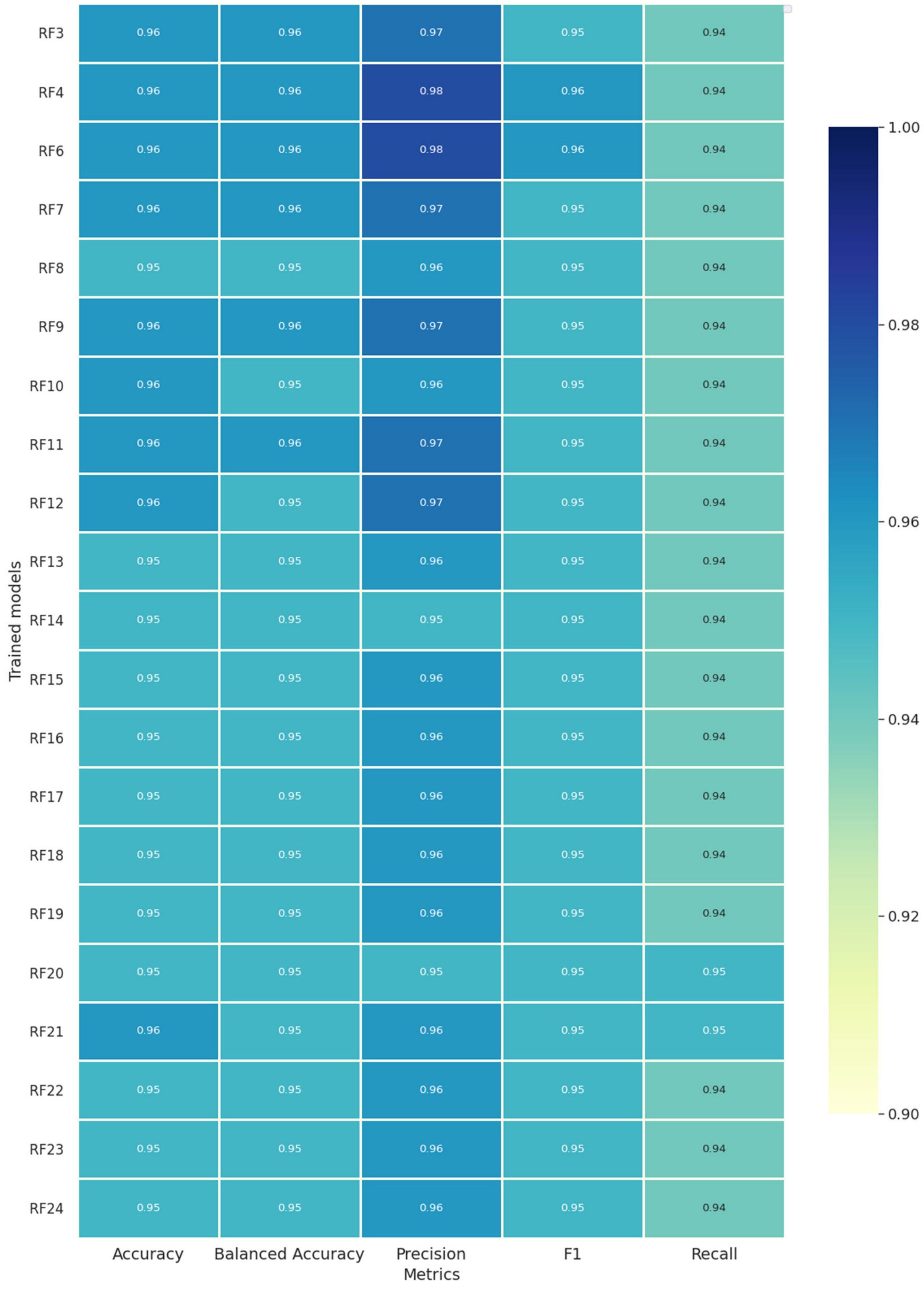

Figure S1. RF metrics heatmap.

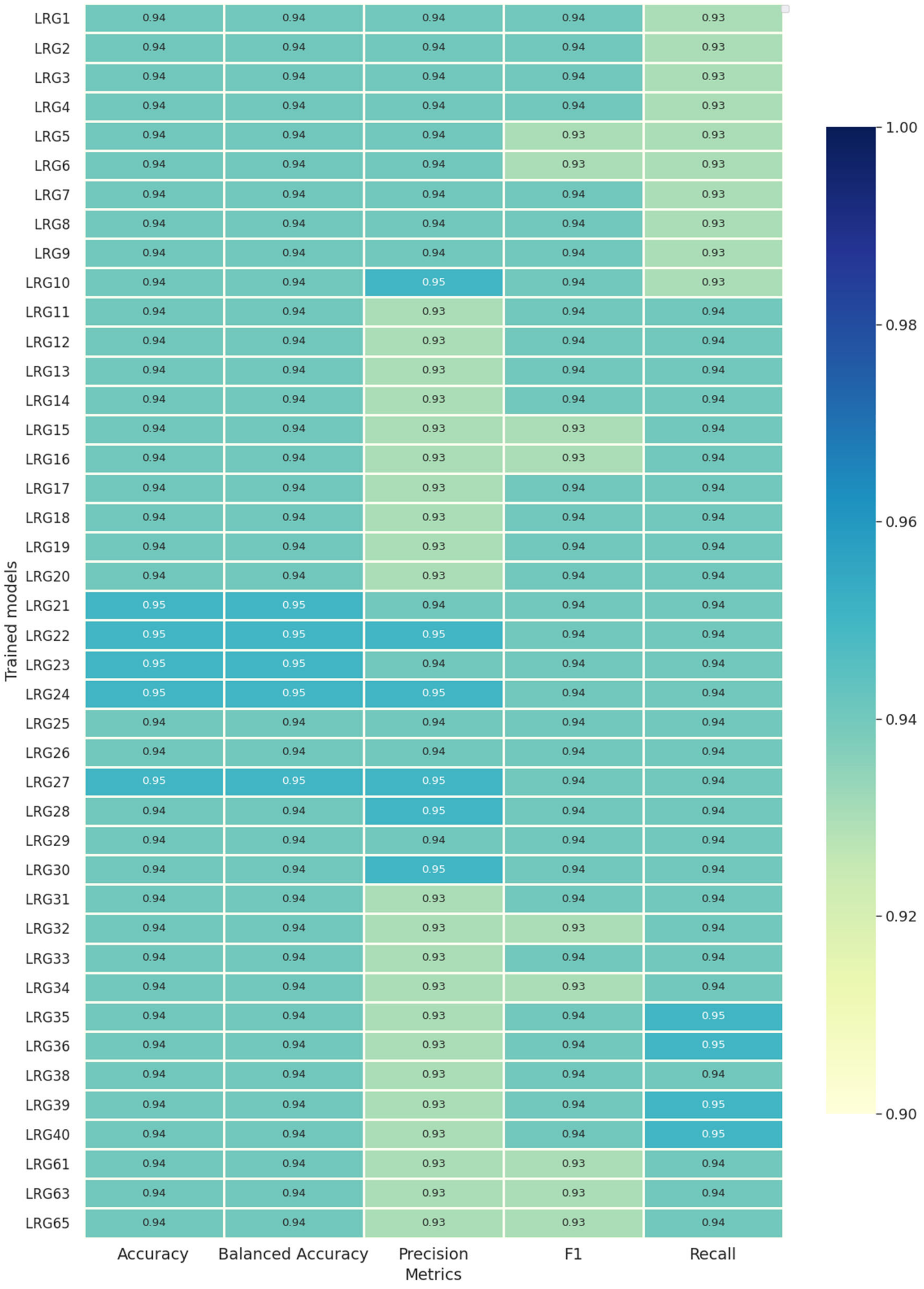

Figure S2. LRG metrics heatmap.

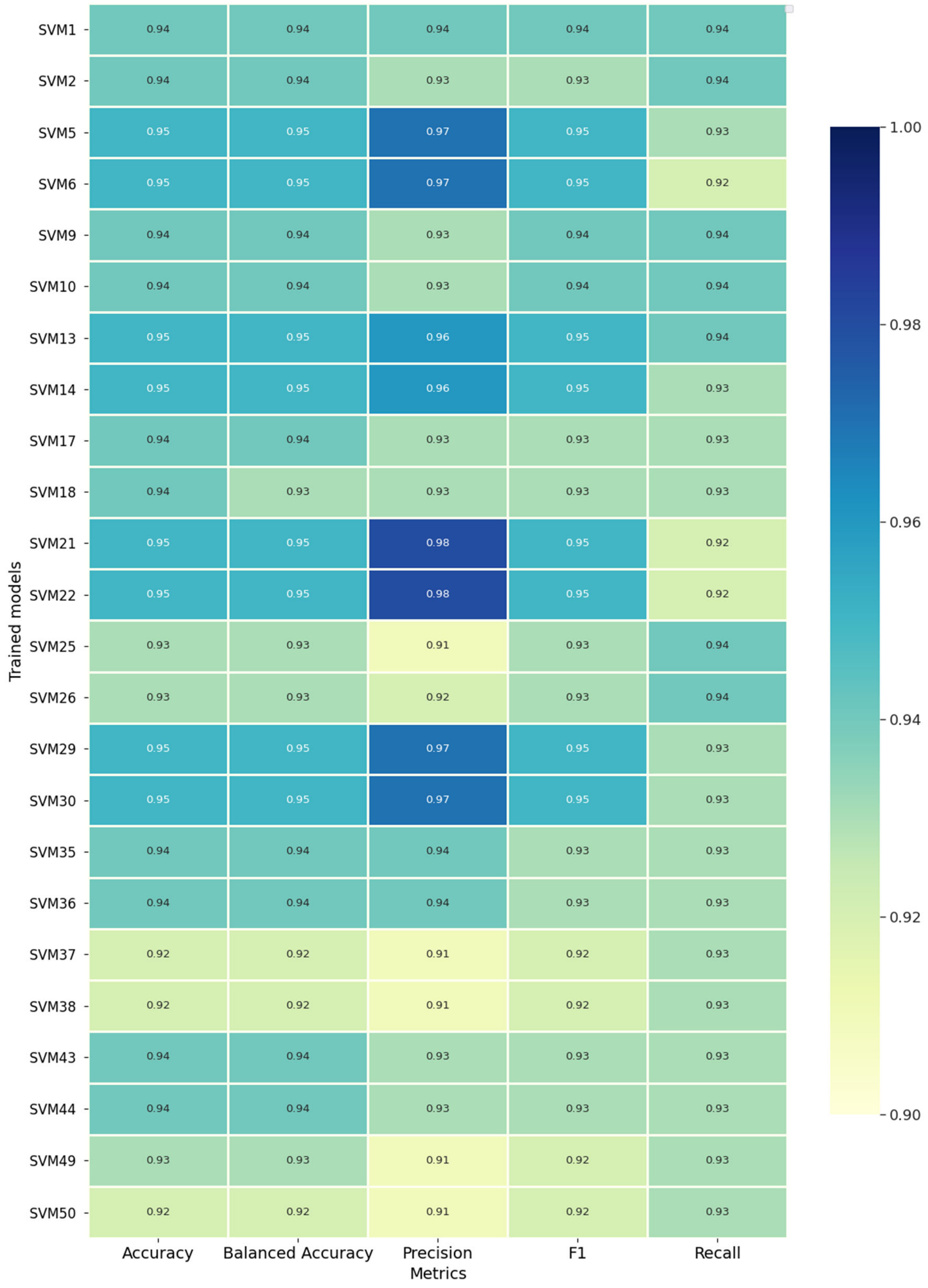

Figure S3. SVM metrics heatmap.
